# Nano-Graphene Oxide Functionalized Bioactive Poly(lactic acid) and Poly(ε-caprolactone) Nanofibrous Scaffolds

**DOI:** 10.3390/ma11040566

**Published:** 2018-04-06

**Authors:** Duo Wu, Archana Samanta, Rajiv K. Srivastava, Minna Hakkarainen

**Affiliations:** 1 Department of Fibre and Polymer Technology, KTH Royal Institute of Technology, SE-100 44 Stockholm, Sweden; duowu@kth.se; 2 Department of Textile Technology, Indian Institute of Technology Delhi, Hauz Khas, New Delhi 110016, India; p.archana.iitd@gmail.com (A.S.); rajiv@textile.iitd.ac.in (R.K.S.)

**Keywords:** PLA, PCL, graphene oxide, scaffold, mechanical properties, biomineralization

## Abstract

A versatile and convenient way to produce bioactive poly(lactic acid) (PLA) and poly(ε-caprolactone) (PCL) electrospun nanofibrous scaffolds is described. PLA and PCL are extensively used as biocompatible scaffold materials for tissue engineering. Here, biobased nano graphene oxide dots (nGO) are incorporated in PLA or PCL electrospun scaffolds during the electrospinning process aiming to enhance the mechanical properties and endorse osteo-bioactivity. nGO was found to tightly attach to the fibers through secondary interactions. It also improved the electrospinnability and fiber quality. The prepared nanofibrous scaffolds exhibited enhanced mechanical properties, increased hydrophilicity, good cytocompatibility and osteo-bioactivity. Therefore, immense potential for bone tissue engineering applications is anticipated.

## 1. Introduction

Bone tissue engineering (BTE) is a promising approach for repairing bone defects. It refers to in situ implantation of a biocompatible/biodegradable scaffold which can be invaded with surrounding cells/tissue, and thus guide tissue regeneration towards new bone formation [1,2,3,4]. Furthermore, scaffolds that possess nanofibrous structure mimic the role of natural extracellular matrix (ECM) and can provide support for cell growth and guide cell behavior [5]. Frequently, poly(lactic acid) (PLA) and poly(ε-caprolactone) (PCL) are utilized as biomedical scaffold materials due to their good biocompatibility and biodegradability. Both of them are Food and Drug Administration-approved polymers and regarded as soft and hard tissue compatible bioresorbable materials [6]. However, PLA and PCL scaffolds have insufficient mechanical strength, limiting their application as bone scaffold in load-bearing applications. In addition, they are typically quite hydrophobic in vivo and possess no bioactivity for bio-mineral growth. Therefore, strategies are needed to manufacture osteo-bioactive PLA and PCL scaffolds with enhanced mechanical properties.

The growth of the electrospinning technique further paved the way for manufacturing nanofibrous scaffolds which resemble the structure of ECM. Electrospinning is now widely considered as a simple and up-scalable means of fabricating ultrafine nanofibers [7,8,9,10]. Electrospun fibrous scaffolds possess high porosity and large surface area to better accommodate cell attachment and proliferation. PLA and PCL have successfully been fabricated into electrospun nanofibrous scaffolds for a variety of applications [11,12,13,14,15,16]. The addition of property-enhancers such as carbon-based nanomaterials [17,18], calcium carbonate [19,20], and hydroxyapatite [19] in neat polymer scaffold is a possible strategy to endow the scaffolds with certain functionalities to meet different requirements. 

Graphene oxide (GO) has recently attracted great research interest in tissue engineering due to its unique physicochemical properties. Two-dimensional (2D) GO has high Young’s modulus, excellent flexibility, and a large number of hydrophilic groups, and thus it is considered as a promising nano-enhancer for bone scaffolds [21,22]. Specially, it can promote hydroxyapatite (HA) mineralization [23,24,25], imparting osteo-bioactivity to the composite scaffolds. Novel zero-dimensional (0D) nano-graphene oxide dots were developed previously in our group [26,27,28]. The production route firstly includes a microwave-assisted hydrothermal carbonization of starch [29] or cellulose [30,31], and secondly oxidation of the carbon residues to lateral dimension nano-sized graphene oxide dots (nGO). In previous works, we have successfully fabricated solution-casted PCL films [32], freeze-dried porous starch scaffolds [33], and electrospun starch nanofibers [34] with incorporated nGO. All of these composites showed high ability of HA mineralization, indicating that nGO has immense potential as a nano-enhancer in tissue engineering scaffolds. In this work, we aimed to prepare PLA/nGO and PCL/nGO functional nanofiber scaffolds through electrospinning technique. Our hypothesis was that nGO, as a promising nano-enhancer, could both strengthen the mechanical properties of PLA and PCL nanofibers and induce HA mineralization. Further, the electrospinnability, hydrophilicity, and cell viability of PLA/nGO and PCL/nGO nanofibrous scaffolds were evaluated.

## 2. Materials and Methods 

### 2.1. Materials

Polylactide (PLA) was from NatureWorks (Minnetonka, MN, USA) PLA 4032D, comprising approximately 2% D-lactide. Poly(ε-caprolactone) (PCL) with number-average molecular weight (Mn) = 80,000 g/mol was purchased from Sigma-Aldrich (Bangalore, India). Dichloromethane (DCM), and dimethylformamide (DMF) (Merck, Mumbai, India) were used as received. The chemicals used for mineralization test were NaCl, NaHCO_3_, KCl, K_2_HPO_4_∙3H_2_O, MgCl_2_-6H_2_O, HCl (1 mol/L), CaCl_2_, Na_2_SO_4_, (HOCH_2_)_3_CNH_2_, and NaN_3_.

### 2.2. Electrospinning

Nano-graphene oxide (nGO) was prepared according to our previous work [28,33,34]. Briefly, starch was carbonized in dilute sulfuric acid by heating in a microwave oven (Milestone UltraWAVE, Milestone Inc., Sorisole, Italy) for 2 h at 180 °C. The obtained carbon spheres were further oxidized in nitric acid for 30 min at 90 °C. nGO was obtained after rotary evaporation of the diluted acidic solution followed by freeze-drying. PLA and PCL were dissolved in DCM/DMF (4/6 *w*/*w*) at 10% *w*/*w* concentration. Different amounts of nGO were added to the PLA or PCL solutions to prepare 0%, 1%, 2.5%, and 5% (nGO/P, *w*/*w*, where P stands for PLA and PCL) solutions. Electrospinning was performed on these solutions to fabricate PLA and PCL fibers with four different nGO concentrations. The fibers were denoted as P fiber, P-nGO1 fiber, P-nGO2.5 fiber, and P-nGO5 fiber, respectively (P stands for PLA or PCL). Each solution was filled in a 2 mL plastic syringe and electrospun at 20 cm distance from the collector, under a flow rate of 0.5 mL/h and a voltage of 23 kV. Fibers were collected on both aluminum foils and glass covers.

### 2.3. Characterizations

#### 2.3.1. Transmission Electron Microscopy (TEM) 

Transmission electron microscopy images were obtained by a HITACHI HT7700 (high-contrast mode). PLA and PLA-nGO1 fibers were collected on holey 400 mesh copper grids (TED PELLA, Inc. Redding, CA, USA).

#### 2.3.2. Fourier Transform Infrared Spectroscopy (FTIR)

FTIR spectra of P, P-nGO1, P-nGO2.5 and P-nGO5 fibers were recorded by PerkinElmer Spectrum 2000 FTIR spectrometer (Norwalk, CT, USA) equipped with attenuated total reflectance (ATR) accessory (golden gate) Grasbey Specac (Kent, UK).

#### 2.3.3. Scanning Electron Microscopy (SEM)

To examine the morphology of all the obtained fibers before and after the cell viability test and mineralization test, an ultra-high resolution field emission (FE)-SEM Hitachi S-4800 was used (Hitachi, Tokyo, Japan). The samples were sputter coated with 3 nm gold layers before analysis. Energy dispersive X-ray spectroscopy (EDS) spectra were acquired on the same Hitachi S-4800 SEM, equipped with an Oxford Instruments X-MaxN 80 EDS (Oxford Instruments, Oxfordshire, United Kingdom) at a voltage of 15kV.

#### 2.3.4. X-ray Diffraction (XRD) 

XRD spectra were recorded by PANalytical X’Pert PRO diffractometer for P, P-nGO1, P-nGO2.5, and P-nGO5 fibers. The X-ray source was CuKR radiation (λ = 0.1541 nm) (PANalytical, Kista, Sweden).

#### 2.3.5. Micromechanical Testing

All the micromechanical measurements were performed on a Deben Microtest tensile tester (Deben UK Ltd, Suffolk, United Kingdom). The collected fiber mats were cut into 10 mm-wide strips and adhered on aluminum foil, which were later mounted in the micromechanical tensile stage. The testing length of the samples was 10 mm. The measurements (five replicates for each sample) were conducted at a strain rate of 0.5 mm/min and the tensile stresses were calculated by dividing the measured force by the thickness of the fiber mat.

#### 2.3.6. Water Contact Angle Measurements 

The water contact angle measurements of P, P-nGO1, P-nGO2.5, and P-nGO5 fiber mats were performed using a contact angle and surface tension meter (KSV Instruments Ltd., Gothenburg, Sweden). A drop of Milli-Q water (5 mL) was placed on the surface of the sample, and images of the water menisci were recorded by a digital camera. The contact angle of each sample was calculated from three measurements at different points.

#### 2.3.7. Cell Viability Test

Cell viability tests were performed to measure the cytotoxicity of all the prepared fibers. Osteoblastic cells MG63 were cultured in Dulbecco’s modified eagle medium (DMEM), 10% heat-inactivated fetal bovine serum (FBS). Each fiber mat on glass cover was placed on the bottom of a 24-well microtiter plate (NUNC A/S, Roskilde, Denmark). The cells were seeded on top of the glass cover in the microtiter plate at a density of 10^4^ cells/well and incubated in DMEM/well (400 μL) for 24 h. Then, 400 μL of 1× resazurin solution in phosphate buffer solution (PBS) was added to each assay well and incubated for approximately 1 h. The absorbance of each well was measured using a microplate reader (FLUOstar OPTIMA, BMG LABTECH) at a wavelength of 560 nm. The cell viability (%) was calculated from 100 × ([*A*]_test_ − [*A*]_PEI_)/[*A*]_control_, where [*A*]_test_, [*A*]_PEI_, and [*A*]_control_ represent the absorbance values of the wells with P-nGO fibers, with polyethylene imine (positive control), and without fibers (negative control), respectively. The absorbance was the average value measured from six wells in parallel for each sample. In addition, the morphology of cells after 4 days of incubation with P-nGO fibers was taken under optical microscopy at 400× and SEM.

#### 2.3.8. Mineralization Test

A simulated body fluid (SBF) was prepared to evaluate if the P-nGO fibrous scaffolds could facilitate mineralization [35]. A conventional SBF (pH = 7.4) was used containing NaCl (7.996 g/L), NaHCO_3_ (0.350 g/L), KCl (0.224 g/L), K_2_HPO_4_·3H_2_O (0.228 g/L), MgCl_2_·6H_2_O (0.305 g/L), HCl (1 mol/L, 40 mL), CaCl_2_ (0.278 g/L), Na_2_SO_4_ (0.071 g/L), and tris(hydroxymethyl)aminomethane (Tris, 6.057 g/L). The concentration of the ions in SBF were Na^+^ 142, K^+^ 5.0, Mg^2+^ 1.5, Ca^2+^ 2.5, Cl^−^ 147.8, HCO_3_^−^ 4.2, HPO_4_^2−^ 1.0, and SO_4_^2−^ 0.5 (mmol/L). These mimic the concentrations of human blood plasma. The P-nGO fiber mats (1 cm in diameter) were immersed in SBF and kept at 37 °C. The SBF was changed every day. After 11 and 24 days of incubation, all the samples were taken out and rinsed with distilled water. After drying at room temperature, the surface of the fiber mats was characterized by optical microscopy and SEM.

## 3. Results and Discussion

PLA and PCL nanofibers with different concentrations of nGO were prepared by electrospinning to obtain P, P-nGO1, P-nGO2.5, and P-nGO5 for each polymer. TEM and FTIR were applied to investigate the interactions between nGO and PLA or PCL. The morphology, structure, and other properties of P-nGO fibers were evaluated, respectively. 

### 3.1. Interaction between PLA and nGO

**Figure 1:**
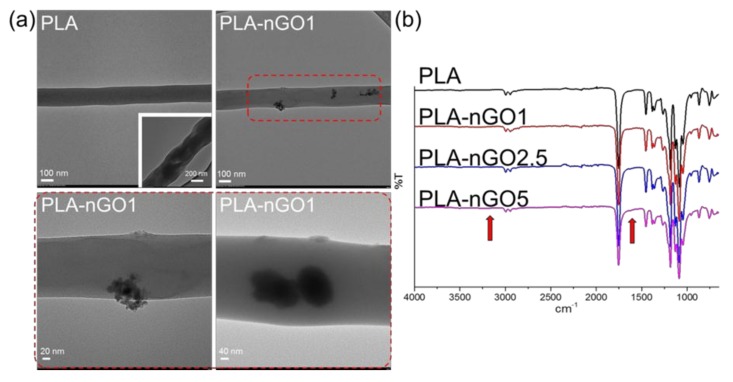
(**a**) TEM images of poly(lactic acid) (PLA) and PLA-nGO1 fibers; (**b**) Fourier transform infrared (FTIR) spectra of PLA, PLA-nGO1, PLA-nGO2.5, and PLA-nGO5 fibers.

Figure 1a shows the TEM images of the PLA fibers and PLA-nGO1 fibers. It can be seen that neat PLA fibers mostly exhibited homogeneous regular morphologies. This is in accordance with most of the previous papers. Some rough fibers were also seen (in the inserted image), probably indicating poor volatility of the solvent. nGO clusters (or agglomerations) were seen in the case of PLA-nGO1 fibers. The enlarged images suggest that nGO could either attach on the surface of PLA fibers or be imbedded inside the PLA fibers. Previously, it was shown that strong intermolecular hydrogen bonds can be formed between the carbonyl groups of polyester and hydrogen-donating groups of graphene oxide [36,37]. 

In the FTIR spectra (Figure 1b), stretching of acidic and phenolic OH, a broad peak from 2500 to 3500 cm^−1^, and stretching of C=C, a shoulder for carbonyl peak at ~1650 cm^−1^, were assigned as originating from the nGO. The intensity of these peaks consequently slightly increased as the concentration of nGO in the composites increased. In summary, Figure 1 illustrates that nGO was successfully incorporated into the PLA electrospun fibers either on the surface or inside the fibers. This indicates favorable secondary interactions between PLA fibers and nGO.

### 3.2. Fiber Morphology

**Figure 2:**
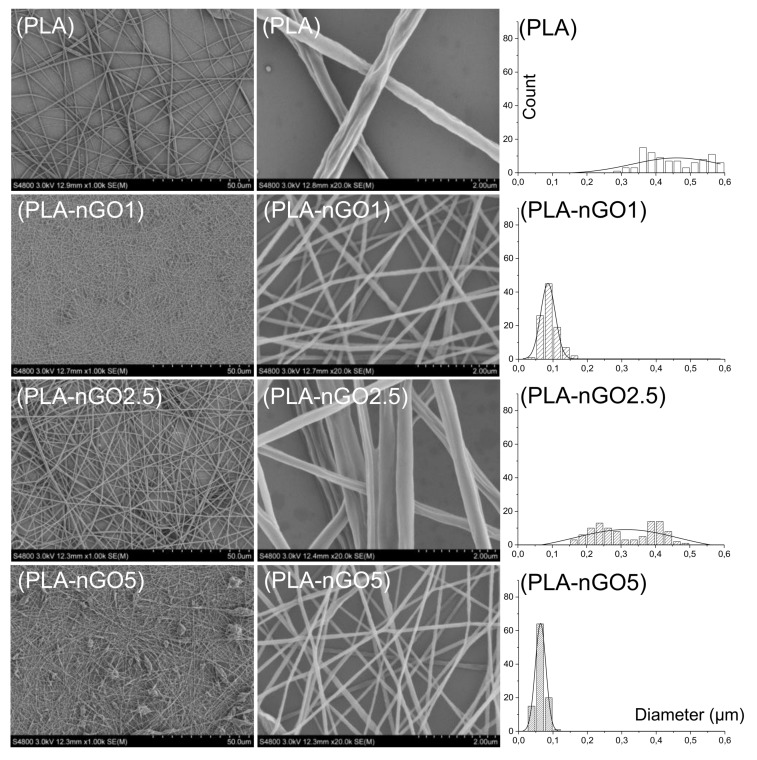
SEM images and the fiber size distribution of PLA, PLA-nGO1, PLA-nGO2.5, and PLA-nGO5 fibers.

SEM was applied to examine the morphology of the prepared PLA-nGO nanofibers (Figure 2). It is clearly shown that nanofibers were successfully prepared through electrospinning of PLA-nGO composite solutions. Our previous study showed that the addition of nGO enhanced the electrospinnability of starch, leading to thinner and more uniform fibers. However, it was also found that high concentration of nGO may cause agglomeration and non-uniformity of the continuous jet, forming more beads [34]. In another work, Yoon et al. reported that the diameter of the electrospun poly(lactide-co-glycolide ) (PLGA)/GO nanofibers was lower than that of pristine PLGA [38]. The SEM images here of the systems containing 0D nGO are in agreement with the previously reported phenomenon for 2D GO systems. The diameter of the neat PLA fiber was in the range of 200–600 nm. A number of beaded defects were found in the PLA-5nGO fibers, while they simultaneously showed the thinnest and most uniform morphology. The addition of nGO also narrowed the size distribution of the fiber diameters. The repulsive forces of the jet sprays during electrospinning depend on the parameters of the polymer solution, such as the electrostatic repulsive charge, conductivity, and viscosity [39,40]. Therefore, the decrease of the fiber diameter could be attributed to the large charge accumulations in the solution jets caused by the abundant charges on the surfaces, leading to strong electrostatic repulsions [41,42]. It is interesting that PLA-nGO2.5 did not follow the trend of decreasing fiber diameter: in this case, the fibers were held in bundles and few beads were detected. A possible reason could be that nGO at this concentration (2.5% *w*/*w* nGO/PLA) dispersed evenly on surface of the fibers, avoiding the non-uniformity of continuous jet (form few beads). Meanwhile the interactions with nGO at this concentration are strong enough to promote fiber coalescence. Earlier, we also observed a different behavior for PCL films with 2.5% nGO as compared to lower and higher concentrations of nGO, which was deduced to be due to differences in agglomeration/self-assembly of nGO at different concentrations [32]. In short, nGO is beneficial for PLA electrospinnability, and high concentration of nGO leads to thinner fibers. However at high nGO concentration there are also more beaded defects. Around 2.5% is a critical concentration for PLA/nGO composite to form few beads and to hold the fiber bundles together.

### 3.3. Fiber Microstructure 

**Figure 3:**
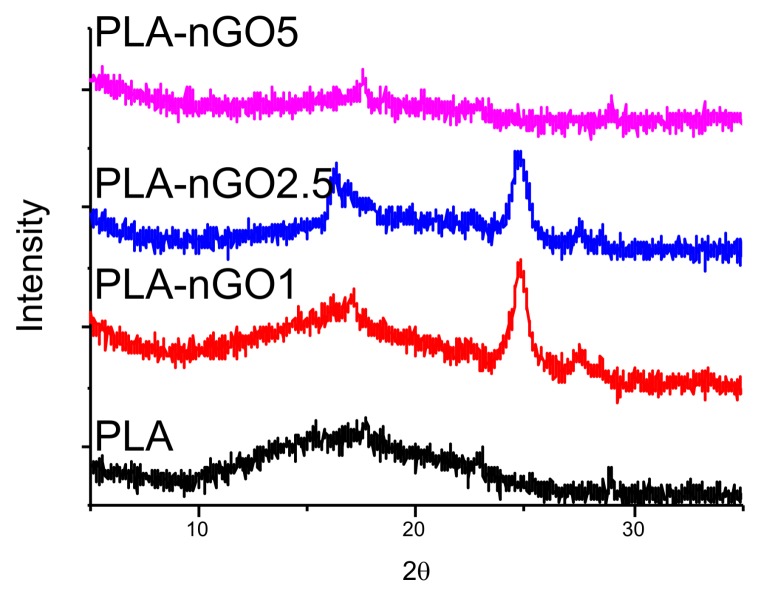
XRD spectra of PLA, PLA-nGO1, PLA-nGO2.5, and PLA-nGO5 fibers.

XRD was conducted to illustrate the microstructure of PLA and nGO (Figure 3). For neat PLA fibers, only a broad scattering reflection located at around 2θ = 16° was detected in the XRD spectrum, indicating that it did not crystallize during the electrospinning process. Figure S1 shows the XRD spectrum of nGO alone. In accordance with the XRD pattern of nGO determined in previous work exhibited a sharp intensity peak at 2θ = 24° (002) is observed [28,33]. This peak is clearly visible in the spectra of most of the PLA-nGO composites. However, PLA-nGO5 is an exception. Possibly, the defected beads in PLA-5nGO shielded the signal intensity of nGO.

After evaluation of morphology and microstructure, the mechanical properties, cell compatibility, and bioactivity of PLA-nGO fibers were investigated by tensile testing, water contact angle measurements, cell viability assay, and mineralization test in SBF.

### 3.4. Mechanical Properties 

**Figure 4:**
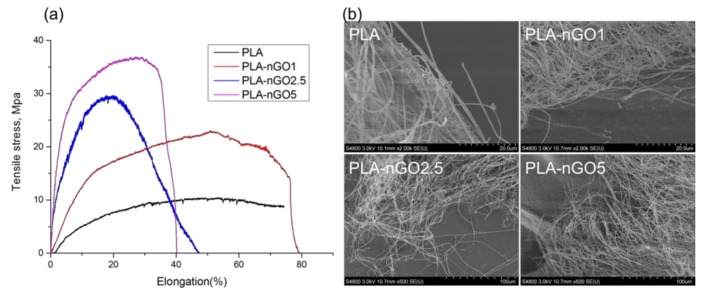
(**a**) Stress-strain curves of PLA, PLA-nGO1, PLA-nGO2.5 and PLA-nGO5 fibers; (**b**) SEM images of PLA, PLA-nGO1, PLA-nGO2.5, and PLA-nGO5 fibers after tensile break.

The mechanical properties of the electrospun fiber mats were investigated by tensile testing. Typical stress–strain curves for the composite fibers are presented in Figure 4a. The test samples were electrospun fiber mats (a combination of randomly distributed single fibers). Each fiber fractured individually during tensile stretching, so there was no abrupt end to stress curve as the strain increased. Instead the stress glided down gradually. A significant improvement of tensile strength and Young’s modulus was achieved for all the PLA-nGO samples as compared to the neat PLA, and the enhancement correlated with the nGO concentration: higher concentration of nGO led to greater enhancement. Because there was no abrupt end to stress curve as the strain increased, it was difficult to define the exact point of elongation at break. The mechanical reinforcement of composites by fillers relies on the effective load transfer from the matrix to the fillers [43], which can be achieved when there are strong interactions at the nanofiller–matrix interface and the nanofillers are dispersed uniformly in the matrix [44]. Therefore, the significant enhancement of strength and modulus also indicates the existence of strong interfacial bonding between PLA and nGO, and uniform dispersion of nGO in PLA fiber. SEM images of the fracture after tensile break gave additional information. Neat PLA fibers were broken sharply during the tensile stretching (short time fracture), PLA-nGO fibers got more aligned and were broken gradually. This offers a chance to control and tune the strength and modulus of the composite scaffolds for a desired application.

### 3.5. Biocompatibility Test 

**Figure 5:**
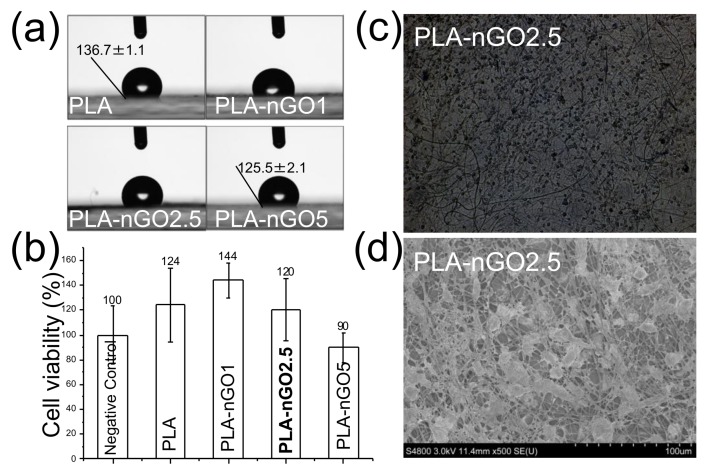
(**a**) Water contact angle measurements on PLA, PLA-nGO1, PLA-nGO2.5, and PLA-nGO5 fibers; (**b**) The relative cell viability of PLA, PLA-nGO1, PLA-nGO2.5, and PLA-nGO5 fibers; (**c**) Optical microscopy images of PLA-nGO2.5 fibers after 4 days of cell culture; (**d**) SEM image of PLA-nGO2.5 fibers after 4 days of cell culture.

Surface wettability is vital to cell attachment and proliferation. Cell attachment on a hydrophilic surface is more efficient as compared to a hydrophobic surface. The water contact angle measurements of PLA, PLA-nGO1, PLA-nGO2.5, and PLA-nGO5 fibers are depicted in Figure 5a. A decreasing water contact angle was observed as nGO loading increased in the PLA-nGO fibers. nGO has plenty of oxygen functionalities, such as –OH and –COOH on its edges. Therefore, a higher loading of nGO can result in higher hydrophilicity. A cell viability test on the surface of PLA fibers and nGO-loaded PLA fibers was performed using osteoblast MG63 cell lines (Figure 5b). Previous study suggested that, under a certain concentration (lower than 1 mg/mL in solution), nGO has no cytotoxicity to MG63 cells [33,34]. However, some negative effects have been observed at high nGO concentration. This was most likely connected to the acidic pH caused by high nGO concentration due to the richness of –COOH groups in nGO. nGO is rich in –COOH groups and a solution with nGO has a concentration dependent acidic pH [45]. Figure 5b shows that PLA, PLA-nGO1, and PLA-nGO2.5 fibers have cell viability higher than 120%, suggesting biocompatibility with MG63 cells. There was a 10% reduction of cell viability on the surface of PLA-nGO5. Optical and SEM images of the MG63 cells on the PLA-nGO2.5 fibers after 4 days of culture were also taken (Figure 5c,d) and MG63 cells could uniformly adhere on the surface of fiber mats with a high density. They attached, grew, and spread along the fibers, exhibiting a normal spindle-like morphology. Additional optical microscopy images of PLA, PLA-nGO1, PLA-nGO2.5 and PLA-nGO5 after cell culture are provided in Figure S2 in supporting information. Cell viability test, thus, clearly indicates that the designed PLA-nGO electrospun fibers are well biocompatible with MG63 cells.

### 3.6. Biomineralization in SBF 

**Figure 6:**
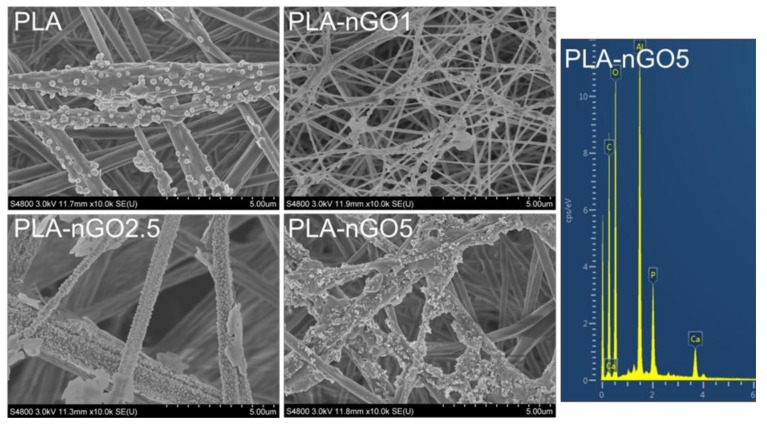
SEM images of PLA, PLA-nGO1, PLA-nGO2.5, and PLA-nGO5 fibers after 11 days of mineralization in simulated body fluid (SBF) and EDS spectra of PLA-nGO5 fibers after 11 days of mineralization in SBF.

**Figure 7:**
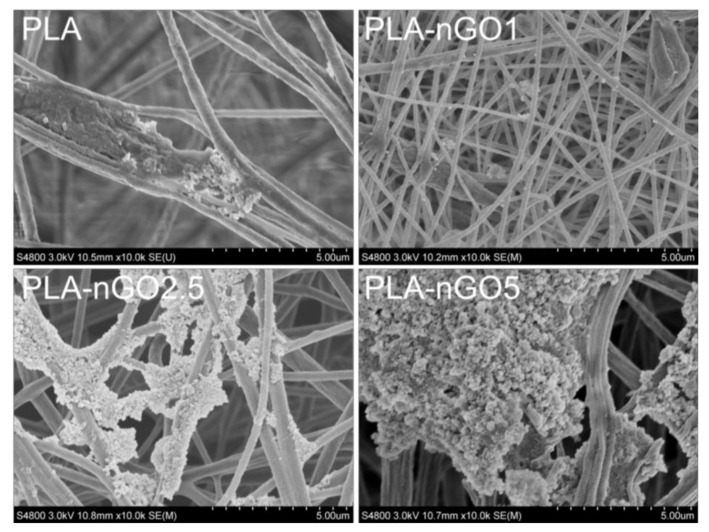
SEM images of PLA, PLA-nGO1, PLA-nGO2.5, and PLA-nGO5 fibers after 24 days of mineralization in SBF.

2D GO has been shown to exhibit the ability to promote hydroxyapatite mineralization [23,46,47]. Our previous study also proved that 0D nGO, after fabrication in PCL films and starch scaffold, could induce mineralization of CaP on the surface of the composites [32,33,34]. In order to evaluate whether nGO could also induce PLA nanofibers’ bioactivity, mineralization tests were performed on the different PLA-nGO fibers. PLA fiber mats were incubated in SBF at 37 °C for 11 and 24 days. SEM/EDS was employed to examine the PLA-nGO fibers and the formed minerals (Figure 6 and Figure 7). Neat PLA fibers possessed only weak capability to induce mineralization due to the relatively high hydrophobicity and lack of nucleation points. Some crystals still appeared, probably due to heterogeneous nucleation of NaCl and CaP minerals [33,34]. The addition of nGO clearly enhanced the amount of minerals that crystallized on the surface of PLA-nGO fibers. The higher the nGO concentration in the PLA-nGO fiber was, the more crystals were induced during the mineralization incubation. Additionally, EDS proved that the formed crystals consisted of CaP. Furthermore, longer incubation time could strengthen the bioactivity and further facilitate the CaP crystal growth on the fiber surfaces (Figure 7). The designed PLA-nGO electrospun fibers were thus capable of inducing CaP crystallization. 

In summary, nGO could be incorporated in PLA electrospun fiber mats. nGO was located either on the surface or imbedded inside of PLA fibers through secondary interactions. nGO improved the PLA electrospinnability, leading to thinner fibers but with more beaded defects. Secondly, nGO altered the macrostructure of PLA fibers. In addition, nGO significantly enhanced the mechanical properties (i.e., the tensile strength and modulus) of PLA fiber mats. Furthermore, PLA-nGO composites were proven to be biocompatible with osteoblast cells MG63 up to a relatively high concentration (1 mg/mL). nGO could induce bioactivity as proven by the mineralization of CaP, creating bioactive functionalized PLA scaffolds. 

### 3.7. PCL-nGO Electrospun Fibers

**Figure 8:**
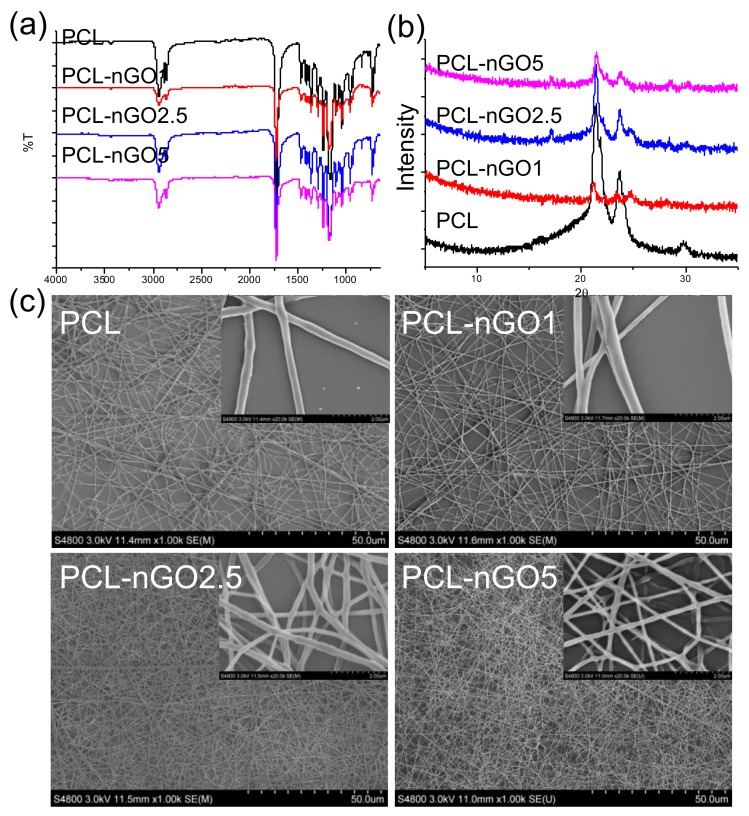
(**a**) FTIR spectra of poly(ε-caprolactone) (PCL), PCL-nGO1, PCL-nGO2.5, and PCL-nGO5 fibers; (**b**) XRD spectra of PCL, PCL-nGO1, PCL-nGO2.5, and PCL-nGO5 fibers; (**c**) SEM images of PCL, PCL-nGO1, PCL-nGO2.5, and PCL-nGO5 fibers.

To investigate whether compositing with nGO is a more universal approach to enhancing fiber quality, mechanical properties, and bioactivity of a scaffold material, PCL—a common biocompatible scaffold material—was also composited with nGO and tested following the same procedure as for PLA. FTIR (Figure 8a) illustrates that nGO was incorporated with PCL electrospun fibers through secondary interactions. The XRD pattern of pure PCL electrospun fibers (Figure 8b) exhibits three diffraction peaks at 2θ = 21.4, 22, and 23.7°, which correspond to the orthorhombic planes (110), (111), and (200). The crystal structure of neat PCL remained after fabrication with nGO. In addition, the peak at 2θ = 24° (which corresponds to (002) of nGO) appears in the spectra of PCL-nGO1 and PCL-nGO2.5 composites. Similar to the results obtained for PLA-5nGO fibers, the signal intensity of nGO (2θ = 24°) weakened in the case of PCL-5nGO. Only a few beads were formed and observed through the SEM examination in Figure 8c. Therefore, PCL generally had better electrospinnability as compared to PLA. Again, the addition of nGO led to thinner and more uniform fibers, confirming the trend observed for PLA-nGO electrospun fibers. 

**Figure 9:**
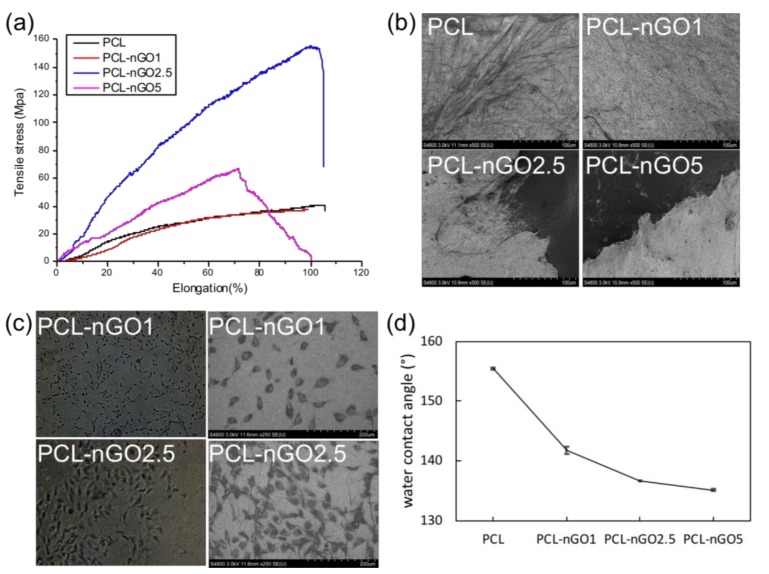
(**a**) Stress–strain curves of PCL, PCL-nGO1, PCL-nGO2.5, and PCL-nGO5 fibers; (**b**) SEM images of PCL, PCL-nGO1, PCL-nGO2.5, and PCL-nGO5 fibers after tensile break; (**c**) Optical microscopy images and SEM images of PCL-nGO1 and PCL-nGO2.5 after 4 days of cell culture; (**d**) Water contact angle measurements on PCL, PCL-nGO1, PCL-nGO2.5, and PCL-nGO5 fibers.

Concerning the mechanical properties (Figure 9a), PCL-nGO fibers showed a slightly different trend than the PLA-nGO fibers. In the case of PLA-nGO, Young’s modulus enhancement correlated with the nGO concentration. However, in the case of the three PCL-nGO fibers, PCL-nGO1 showed more or less similar strength as the neat PCL, PCL-nGO2.5 had significantly enhanced modulus and strength, while PCL-nGO5 exhibited smaller improvement than PCL-nGO2.5. It is believed that nGO structure can reinforce modulus after compositing with polymer. The falling mechanical strength in the case of PCL-nGO5 could be due to the thinner fibers, too-high concentration of nGO, or self-assembly of nGO, in accordance with a previous report [32]. Additionally, the fiber diameter of PCL and PCL-nGO1 was relatively large. They did not completely break off after tensile failure (Figure 9b). However, the materials with thin fibers (i.e., PCL-nGO 2.5 and PCL-nGO5) slit sharply during tensile break. 

Figure 9c shows typical optical microscopy images of PCL-nGO1 and PCL-nGO2.5 after 4 days of cell culture. MG63 cells adhered on the surface of the fiber mats, exhibiting spindle-like healthy morphologies. The images indicate that PCL-nGO fibers were biocompatible with MG63 cells.Additional optical microscopy images of MG63 cells on PCL, PCL-nGO1, PCL-nGO2.5 and PCL-nGO5 can be seen in Figure S3 in supporting information. Furthermore, water contact angle measurements (Figure 9d) showed that higher loading of nGO resulted in more hydrophilic surface, which is expected to promote the biocompatibility.

**Figure 10:**
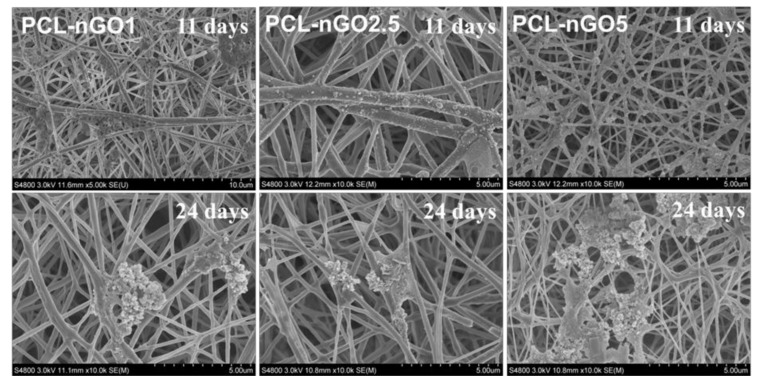
SEM images of PCL-nGO1, PCL-nGO2.5, and PCL-nGO5 fibers after 11 and 24 days of mineralization in SBF.

PCL-nGO fiber mats were incubated in SBF at 37 °C for 11 and 24 days for mineralization test. As shown in Figure 10, the PCL-nGO fibers generally induced less biomineralization on their surface as compared to the PLA-nGO fibers (Figure 6 and Figure 7). This is probably due to the even more hydrophobic nature of PCL fibers (as also supported by the water contact angle measurements). Higher nGO concentration in PCL-fibers induced more crystals after mineralization incubation. In addition, with longer incubation time, more crystals were accumulated and tended to appear in clusters.

In summary, similar to the PLA-nGO electrospun fibers, nGO could enhance the mechanical properties and induce bioactivity for PCL fibers. 

## 4. Conclusions

PLA and PCL nanofibers with incorporated nGO could be successfully fabricated through electrospinning technique. nGO could tightly attach on the surface and inside the fibers. In both PLA and PCL composites, nGO could improve the electrospinnability, enhance the mechanical properties, and impart osteo-bioactivity. At the same time, the biocompatibility of PLA and PCL was retained. Therefore, nGO as a nanoenhancer offers a versatile and convenient way to stronger and bioactive nanofibrous scaffolds for bone tissue engineering applications.

## Supplementary Materials

The following are available online at http://www.mdpi.com/1996-1944/11/4/566/s1.
