# Supplementary material for: Nano-Graphene Oxide Functionalized Bioactive Poly(lactic acid) and Poly(ε-caprolactone) Nanofibrous Scaffolds
Source: Materials (Basel). 2018 Apr 6;11(4):566. doi: 10.3390/ma11040566 (PMC5951450; doi:10.3390/ma11040566)
Supplement: Supplementary file 1 [file materials-11-00566-s001.pdf]

Supplementary information

Nano-Graphene Oxide Functionalized Bioactive Poly(lactic acid) and Poly(ε-caprolactone) Nanofibrous Scaffolds

Duo Wu [1], Archana Samanta [2], Rajiv K. Srivastava [2] and Minna Hakkarainen [1,*]

[1] Department of Fibre and Polymer Technology, KTH Royal Institute of Technology, SE-100 44 Stockholm, Sweden; duowu@kth.se
[2] Department of Textile Technology, Indian Institute of Technology Delhi, Hauz Khas, New Delhi 110016, India; p.archana.iitd@gmail.com (A.S.); rajiv@textile.iitd.ac.in (R.K.S.)
* Correspondence: minna@kth.se; Tel.: +46-8-790-8271

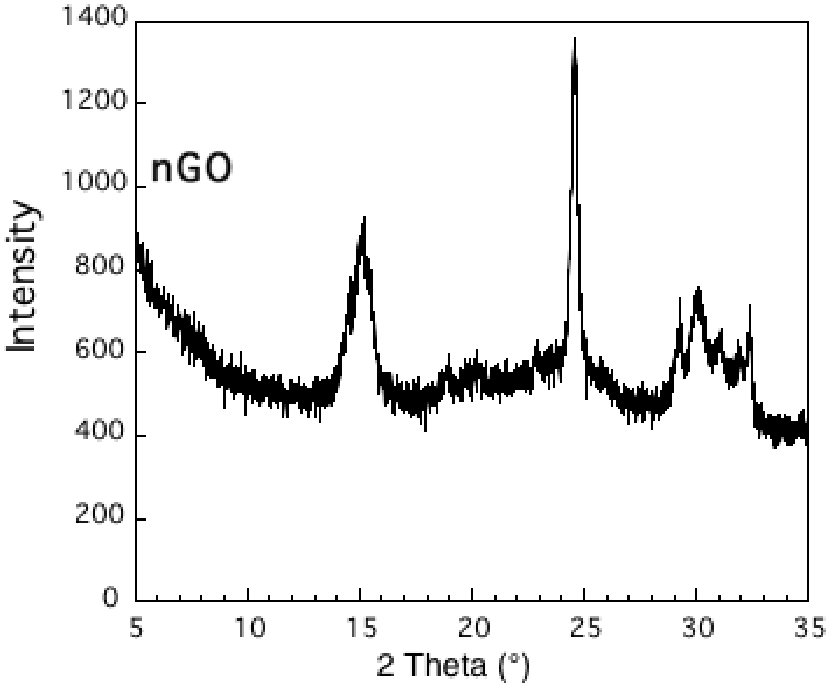

Figure S1. XRD spectrum of nGO

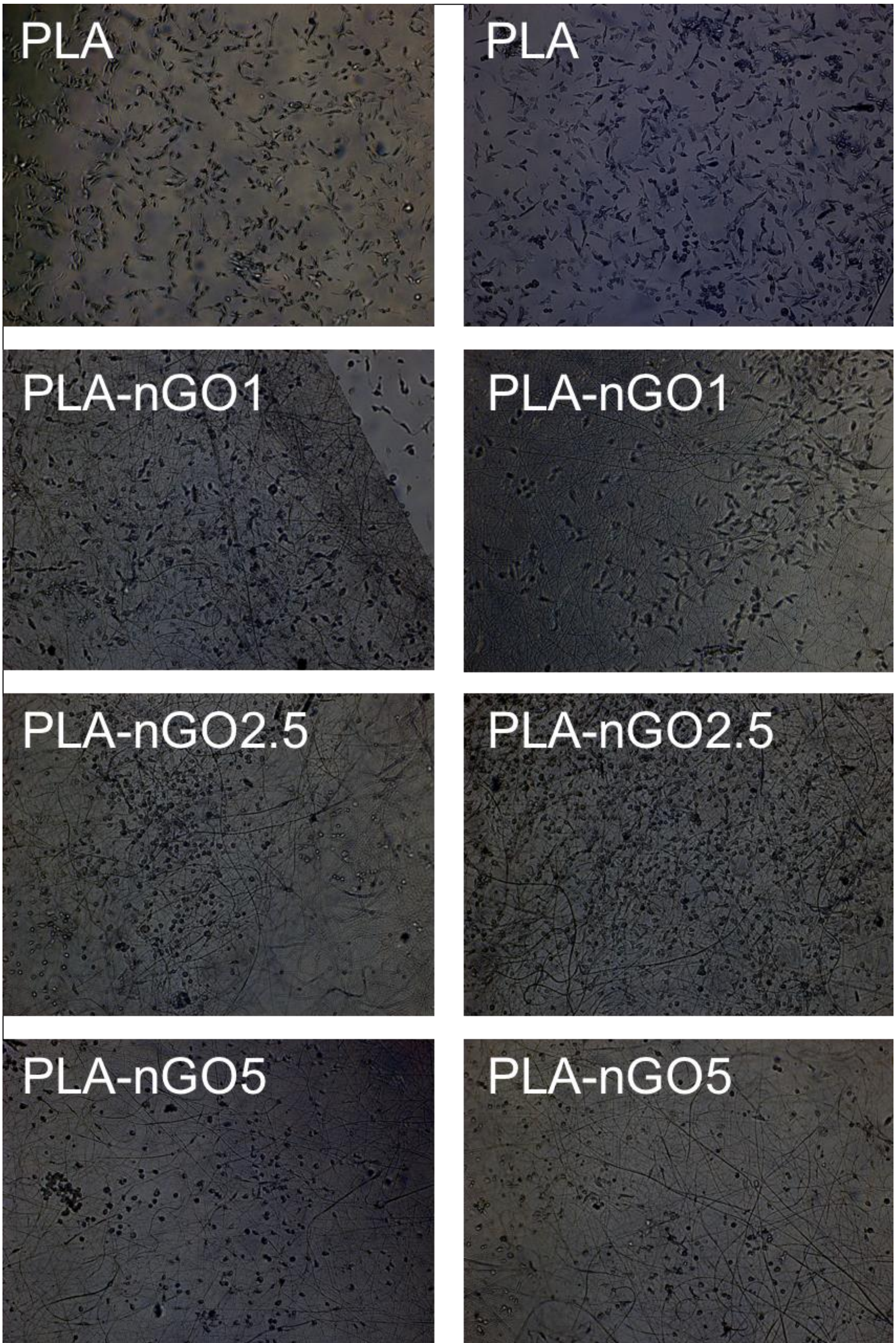

Figure S2. Optimal images MG63 cells on the PLA, PLA-nGO1, PLA-nGO2.5 and PLA-nGO5 after 4 days of culture.

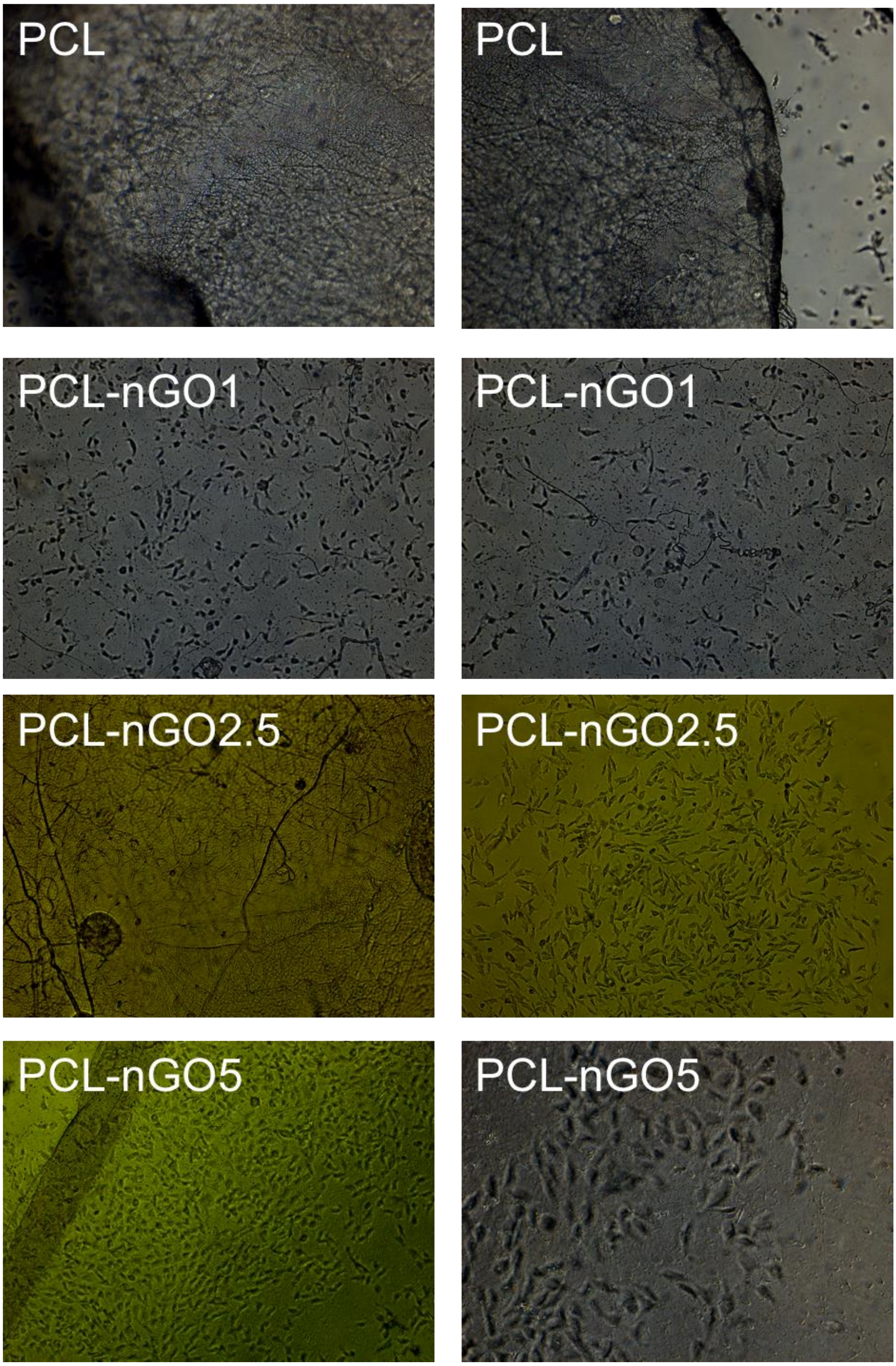

Figure S3. Optimal images MG63 cells on the PCL, PCL-nGO1, PCL-nGO2.5 and PCL-nGO5 after 4 days of culture.
